# Toxin A and B genes expression of *Clostridium difficile* in the sub-minimum inhibitory concentration of clindamycin, vancomycin and in combination with ceftazidime

**Published:** 2020-02

**Authors:** Mohammad Moradi, Shahla Mansouri, Nouzar Nakhaee, Farhad Sarafzadeh, Ebrahim Rezazadeh Zarandi

**Affiliations:** 1 Department of Microbiology and Virology, School of Medicine, Kerman University of Medical Sciences, Kerman, Iran; 2 Department of Community Medicine, School of Medicine, Kerman University of Medical Sciences, Kerman, Iran; 3 Department of Infectious Diseases, Afzalipour Hospital, Kerman University of Medical Sciences, Kerman, Iran; 4 Immunology of Infection of Diseases Research Center, Research Institute of Basic Medical Sciences, Rafsanjan University of Medical Sciences, Rafsanjan, Iran

**Keywords:** *Clostridium difficile*, Antibiotics, Gene expression, Toxin

## Abstract

**Background and Objectives:**

Antibiotics prescribed for infections have diverse effects on microbiota and the pathogen *Clostridium difficile (C. difficile)* as the most important antibiotic-associated diarrhea. This study aims to determine the gene expression of toxins A and B at the transcription level in the sub-MIC of vancomycin (VAN), clindamycin (CLI), and ceftazidime (CAZ) alone and in combination.

**Materials and Methods:**

The MIC and fractional inhibitory concentration (FIC) of two *C. difficile* samples (a clinical isolate and ATCC 9689) were determined by microdilution and checkerboard microdilution methods, respectively. The total RNA was extracted from the medium inoculated with ∼10^6^ CFU/mL of fresh bacteria in the pre-reduced medium containing ½ MIC of antibiotics alone and ½ FIC of antibiotics in combination. Real-time PCR was performed by sybrGreen methods in triplicate, and the data were analyzed by the comparative ΔΔ^CT^ method.

**Results:**

All antibiotics except CAZ (alone and in combination) decreased the gene expression of toxins A and B within 24 hours. VAN and CLI reduced toxin gene expression within 24 and 48 hours. However, CAZ alone and in combination with VAN as well as CLI increased the gene expression of toxins A and B.

**Conclusion:**

The results confirmed toxin gene transcription and toxin production are associated with the type of isolates and antibiotics, as well as the combined form of antibiotics. This could be the reason which can explain the occurrence of *C. difficile* infection among patients who were treated with the third generation of cephalosporins alone and in combination with another antibiotic in the form of combinational therapy.

## INTRODUCTION

*Clostridium difficile*-associated diarrhea (CDAD) is prevalent in hospitalized patients treated with antimicrobial agents in long term periods (1). After the establishment of *Clostridium difficile (C. difficile)* infection (CDI), toxins A and B are produced, which are important virulence factors in the pathogenesis of this bacterium. The toxins with inactivating cellular factors (the Rho family of GTPases, including Rac1 and Rho) disrupt the cell skeleton of epithelial cells; thereby, leading to the occurrence of diarrhea (2). The role of toxins A and B differs in the pathogenesis of *C. difficile*, and it is now verified that toxin B is more important than toxin A in this process (3, 4). Toxins A and B, as well as their regulatory genes, are located in the part of the chromosome called pathogenicity locus (PaLoc) (5). Toxin production occurs in the late exponential and mainly in the stationary growth phases. Certain nutritional factors, including glucose concentration, amino acids, and biotin lead to the upregulation of toxin genes (6). Among other factors, oxidation potentials, temperature variations, genetic properties, and antibiotics regulate toxin gene expression and production in *C. difficile* (7).

Apart from the disruption of microbiota and the consequent induction of CDI, antibiotics exert diverse effects on growth, colonization, gene expression, and toxin production in *C. difficile*. Antibiotics change the pattern of gene expression and toxin production in *C. difficile* in sub-MIC. The sub-MIC effects of various antibiotics, such as VAN, CLI, CAZ, metronidazole, tigecycline, fidaxomicin, linezolid, amoxicillin, and cefoxitin have been investigated on virulence factors in the logarithmic and stationary phases of the growth curve under laboratory conditions and in few cases in a mouse model (8–11).

The combination therapy of infections increases the risk of CDAD in hospitalized patients (12, 13). In our previous study, VAN and CLI alone, as well as in combination with CAZ showed diverse effects on toxin production in *C. difficile*. In addition, VAN, CLI and CAZ alone and in combination inhibited toxin production in 24 hours. After 48 hours, VAN and CLI continued their inhibitory effects on toxin production but did not allow the toxins to be synthesized or released into the medium. In contrast, CAZ alone and in combination ones enhanced toxin production (10).

The presence of *C. difficile* toxins in the medium is generally related to the expression of toxins A and B at the level of transcription. Therefore, this study aims to determine any compatibility mode between the gene expression of toxins A and B and the production of toxins A and B at ½ × MIC of VAN and CLI alone, as well as in combination with CAZ in two *C. difficile* A+/B+/CDT- toxin production types.

## MATERIALS AND METHODS

***C. difficile*** **isolates, MIC, and FICi determination.** A clinical isolate of *C. difficile* was taken from a diarrhea sample. An ATCC 9689 strain was provided from the Microbial Bank of Kerman University of Medical Sciences. The clinical isolate and the ATCC 9689 strain were confirmed as *C. difficile* by the latex agglutination test (Microgen® *C. difficile*, UK) (14). The presence of *tcdA* and *tcdB* genes, as well as the toxin production ability, were established based on previous studies (15, 16). The MIC of antibiotics alone [VAN, CLI, and CAZ (all Sigma, USA)] and the FIC index (FICi) of the combinations (VAN plus CAZ and CLI plus CAZ) were determined for the two tested bacteria by the microdilution and checkerboard microdilution methods as already prescribed (10, 17, 18).

**RNA extraction and cDNA synthesis.** Approximately 106 CFU of fresh bacteria (an 18-hour culture) were inoculated to the tubes with 5 mL of pre-reduced media containing ½ × MIC of VAN, CLI and CAZ alone, as well as ½ × FICi of VAN plus CAZ, and CLI plus CAZ (Table 2). The tubes were incubated in an anaerobic jar (Merck, Germany) at 37°C. After two periods of 24 and 48 hours, 3 ml of the cultures were taken and centrifuged at 10000 × g/3 min. The pellets were washed twice in the TE buffer (Tris-HCL EDTA, pH=7) and used for RNA extraction by the Innupure RNA extraction kit (Gene analytical, Germany) (19). The quality and quantity of the extracted RNA were confirmed using gel electrophoresis and a Nanodrop, respectively. In addition, DNase treatment and cDNA synthesis were performed as previously described (20).

**Table 1.** Primers used for real-time PCR

**Gene**	**Primer**	**Sequence**
*tcdA*	*tcdA*-F	5′'-CAACACCTTAACCCAGCCATA-3′
	*tcdA*-R	5′-AGAGTTTTCTGCGGTAGCTGA-3′
*tcdB*	*tcdB*-F	5′-ATCTGGAGAATGGAAGGTGGT-3′
	*tcdB*-R	5′-TGATGGTGCTGAAAAGAAGTG-3′
*16S* rRNA	*16S*-F	5′-AGCGGTGAAATGCGTAGATAT-3′
	*16S*-R	5′-CGACGTCAGTTACAGTCCAGA-3′

**Table 2.** ½ × MIC and ½ × FIC of two *C. difficile* toxin production types

***C. difficile***	**MIC (μg/mL)**			**FIC (μg/mL) + (μg/mL)**	

	**VAN**	**CAZ**	**CLI**	**VAN plus CAZ**	**CLI plus CAZ**
Clinical isolate	0.25	8	0.5	0.062 + 8	0.5 + 8
ATCC 9689	0.25	8	1	0.062 + 8	0. 5 + 8

**Real-Time PCR.** The gene expression ratios of toxins A and B were performed by the SYBR Green qPCR method and the primers listed in Table 1. The ΔΔ^CT^ of control genes (16S rRNA) and that of target genes (*tcdA* and *tcdB*) were analyzed and represented as fold change by the formula 2^ΔΔCt^. Based on the physiological changes which affect the growth of *C. difficile*, relative expression ratios would be considered significant if relative changes in gene expression were more or less than 1–3 fold (19, 21).

## RESULTS

**MIC and FIC concentrations.** 0 2 demonstrates MIC and FIC concentrations for a clinical isolate and ATCC 96889 strain.

**Fig. 1.**
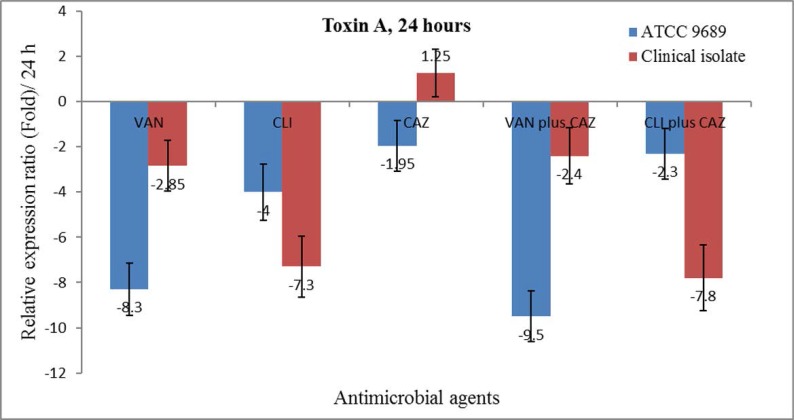
The relative expression ratio of *tcdA* in the ½ × MIC of VAN, CLI and CAZ alone as well as in the combination (½ × FIC) of CAZ plus VAN or CLI in the clinical isolate and the ATCC 9689 strain after 24 hours from incubation under anaerobic conditions.

**Toxin A gene expression.** All antibiotics except CAZ, alone and in combination, reduced toxin A gene expression after 24 hours from incubation. The decline in toxin A gene expression varied between ½ × MIC of antibiotics alone and in combination, with the variations observed between the ATCC 9689 strain and the clinical isolate. The maximum decrease was observed in the VAN plus CAZ combination. CAZ alone did not alter the gene expression of toxin A after 24 hours from incubation (Fig. 1).

**Fig. 2.**
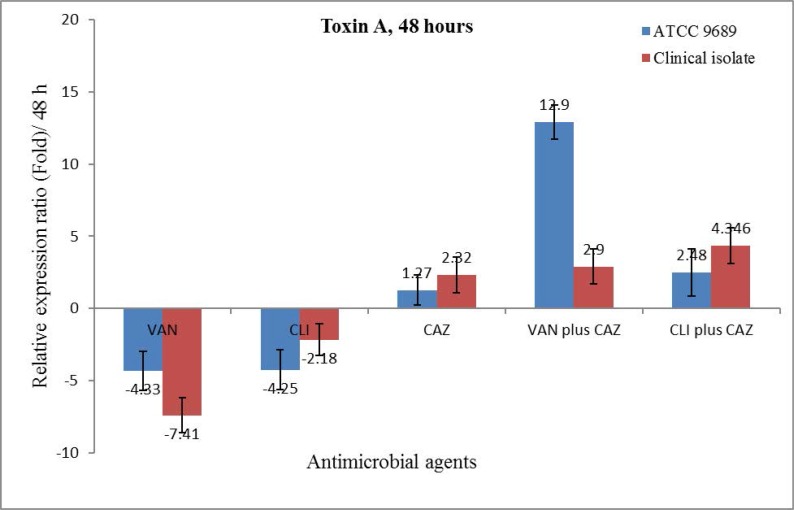
The relative expression ratio of *tcdA* in the ½ × MIC of VAN, CLI, and CAZ alone as well as in the combination of CAZ plus VAN or CLI in the clinical isolate and the ATCC 9689 strain after 48 hours from incubation under anaerobic conditions.

The gene expression level of toxin A was different after 48 hours in comparison with that of after 24 hours. VAN decreased the gene expression ratio of toxin A significantly. CLI reduced toxin A gene expression but was not significant in the clinical isolate. In total, the level of toxin A gene expression in the ½ MIC of VAN was less in 48 hours than in 24 hours. CAZ did not change the level of toxin gene expression in either 24 or 48-hour period. Notably, the expression levels were different in the ½ FIC of CAZ plus VAN or CLI, for the levels depended on the isolate and the type of antibiotic combination (Fig. 2).

**Fig. 3.**
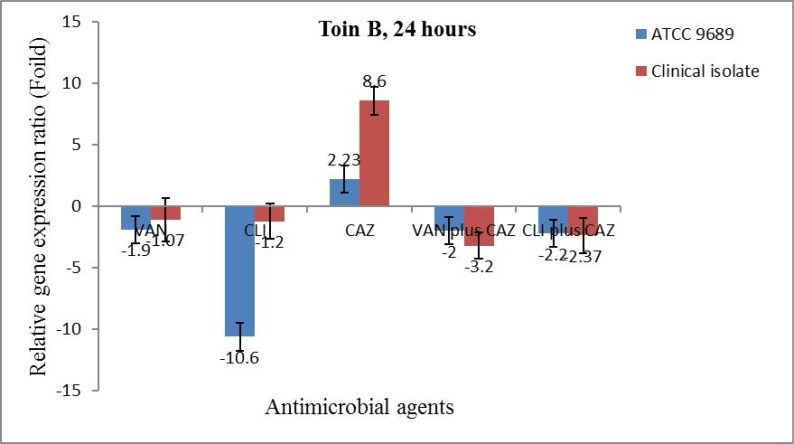
The relative expression ratio of *tcdB* in the ½ × MIC of VAN, CLI and CAZ alone as well as in the combination of CAZ plus VAN or CLI in the clinical isolate and the ATCC 9689 strain after 24 hours from incubation under anaerobic conditions. Foild should be changed to Fold in this picture

**Fig. 4.**
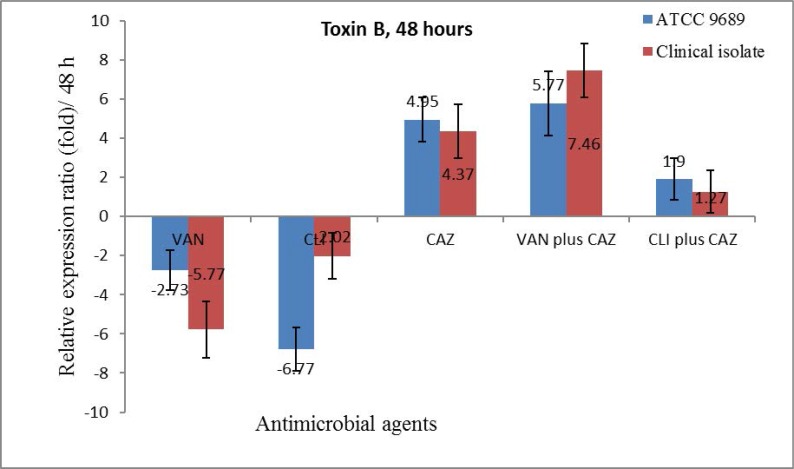
The relative expression ratio of *tcdB* in the ½ × MIC of VAN, CLI and CAZ alonealso in the combination of CAZ plus VAN or CLI in the clinical isolate and the ATCC 9689 strain after 48 hours from incubation under anaerobic conditions.

**Toxin B gene expression.** Toxin B gene expression decreased in the ½ × MIC of all antibiotics alone except CAZ as well as in the combination forms. Nonetheless, the levels of decrease were not significant except in the ½ × MIC of CLI in the ATCC 9689 strain. CAZ upregulated toxin B expression in the ATCC 9689 strain and in the clinical isolate, but it was only significant in the clinical isolate (Fig. 3). The gene expression ratio of toxin B in both the ATCC 9689 strain and the clinical isolate decreased in the ½ × MIC of CLI and VAN, but it increased in the ½ × MIC of CAZ and in the combination of CAZ with VAN and CLI. The decrease in the expression ratio was significant in VAN (the clinical isolate) and in CLI (the ATCC 9689 strain), and the increase was significant in CAZ as well as in combination with VAN. However, the gene expression level of toxin B was insignificant in CLI plus CAZ (Fig. 4).

## DISCUSSION

According to the results, the antibiotics tested, except CAZ alone and in combination, downregulated toxin A and B genes within 24 hours at ½ × MIC. Notably, CAZ did not influence toxin gene expressions. The expression was close to those of normal conditions (the medium without antibiotics). The down expression of toxin A and B genes in the ½ × MIC of VAN and CLI continued for the next 48 hours. During this time, *C. difficile* upregulated the gene expression of toxins A and B although they were still close to normal conditions. Overexpression was primarily observed in the ½ × MIC of CAZ and secondarily in combination with CLI and VAN. The data revealed a positive correlation between toxin A and B gene expression and toxin production (the previous study) (10). However, there were some differences in the level of toxin gene expression.

CAZ, as a member of third-generation cephalosporins, is an antibiotic prescribed for the treatment and prevention of various infections in hospitalized patients (22). The expression ratio of toxin A and B genes in the sub-MIC of CAZ was similar to the ones in control medium. In our previous study, in contrast to the control medium, *C. difficile* did not produce toxins in the presence of CAZ within the 24-hour period. It is worth mentioning that the RNA extracted from the same medium was used for the toxin assay (10). It implies the notion that the clinical and ATCC 9689 strains of *C. difficile* have not reached the late exponential or stationary phases (23). Therefore, the toxins might not have been synthesized or secreted into the medium. Upon an increase in the incubation time (48 hours), *C. difficile* adapts itself to the new conditions (the existence of antibiotics) and is less affected by CAZ (20). As a result, *C. difficile* continues toxin production at the presence of the sub-MIC of CAZ as a cephalosporin. It will probably lead to the accumulation of toxins in the colon *in vivo* and especially in pseudomembranous colitis; thereby, causing more damage to the intestinal epithelial cells.

Upon an increase in the incubation period upto 48 hours, the combination of CAZ with CLI and VAN had an effect similar to that of CAZ. CAZ neutralizes the downregulatory effects of VAN and CLI in combination. In the previous study, the toxins in combination were more effective than CAZ alone (10). The enhancement in toxin production is observed in the gene expression of toxins A and B in this study. It has been reported that the combination therapy increases the incidence of CDAD (12, 13). The incidence of CDAD in patients receiving a combination therapy has been less investigated (12). Hence it seems necessary to investigate the effects of combination therapy on the incidence of CDI as well as the level of toxins in the colon in the hospitalized patients.

It appears that a positive correlation exists between toxin gene expression and the level of toxin production. *C. difficile* infection occurs in patients who receive antibiotics (10). The bacterium may be influenced by antibiotics, but in the end, it will adapt itself to new conditions and the expression of toxin genes as well as toxin production reach natural conditions and even higher.

## CONCLUSION

The level of toxin A and B production *in vitro* and probably *in vivo* (the colon) is associated with the isolate and the type of antibiotics. Some antibiotics, apart from disrupting microbiota, downregulate toxin gene expression (CLI and VAN), and some others (CAZ alone and in combination with CLI and VAN) upregulate toxin A and B genes. Apparently, one of the reasons for the overproduction of toxins is theiroverexpression(AandB)atthepresenceofthe sub-lethal concentration of antibiotics, such as CAZ alone or in combination with CLI and VAN. The results confirmed a positive correlation between toxin gene expression and toxin A and B production.
